# Supplementary figures and images for: Maternal and neonatal IgG against Klebsiella pneumoniae are associated with broad protection from neonatal sepsis: a case-control study of hospitalized neonates in Botswana
Source: medRxiv. 2024 May 28:2024.05.28.24308042. Preprint. [Version 1] doi: 10.1101/2024.05.28.24308042 (PMC11160826; doi:10.1101/2024.05.28.24308042)

Figure S1

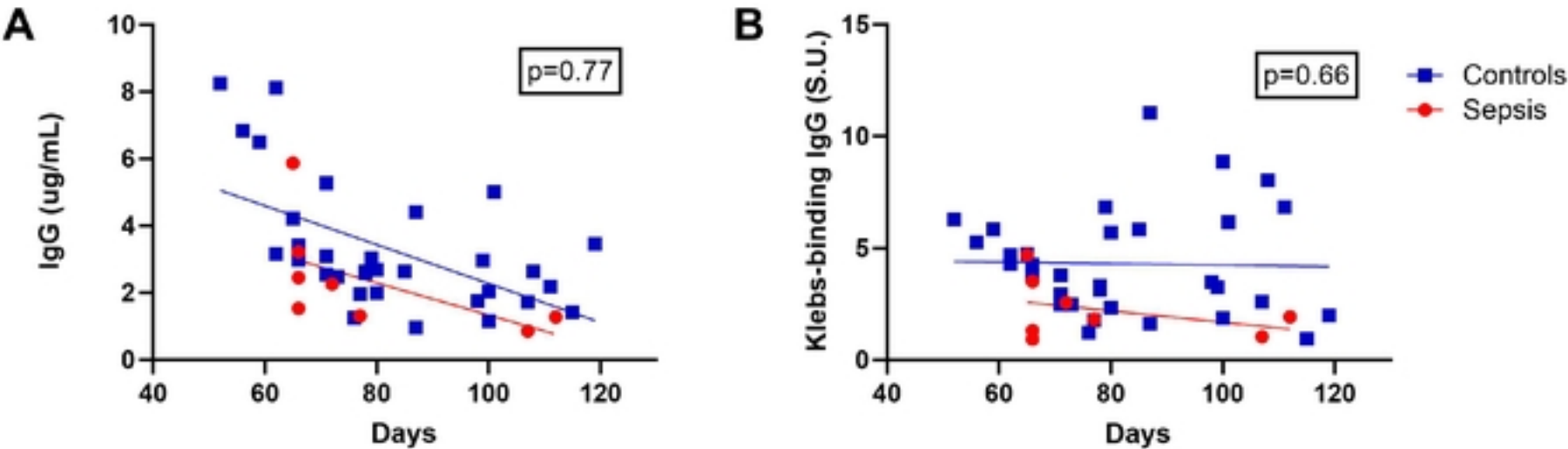

Fig S1

Figure S2

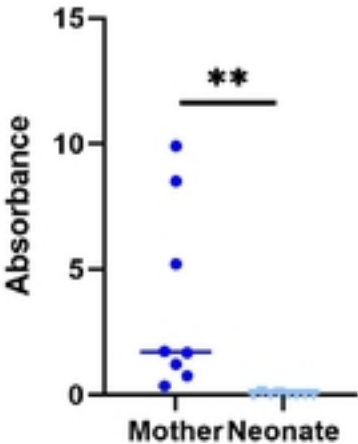

Fig S2

Figure S3

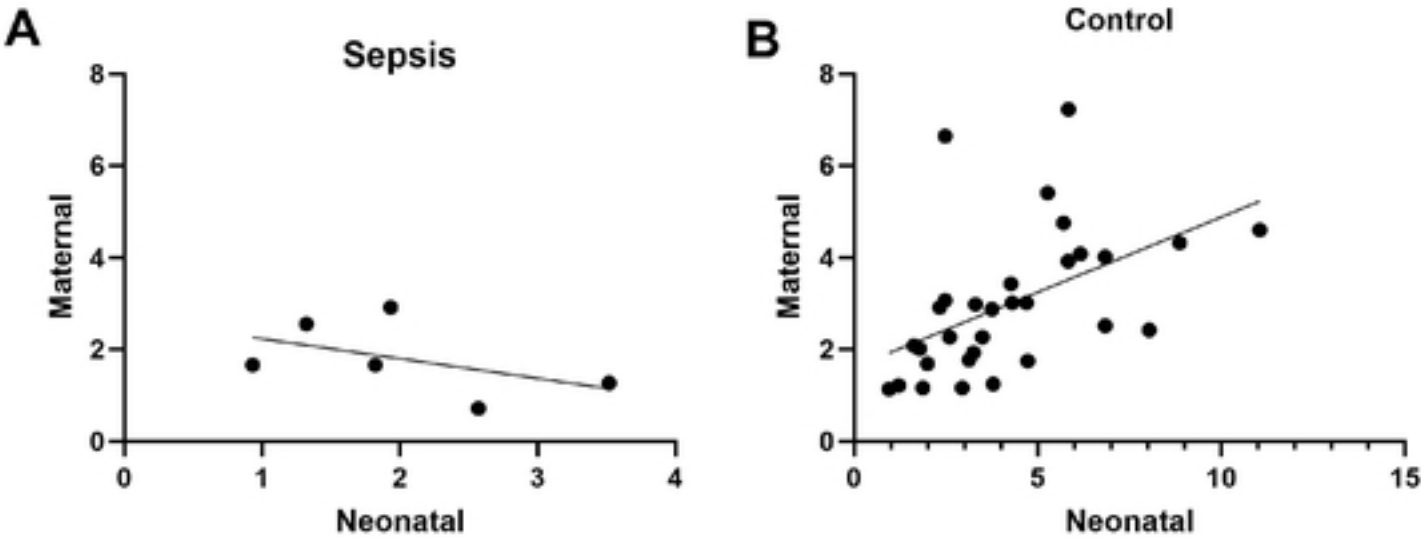

Fig S3

Figure S4

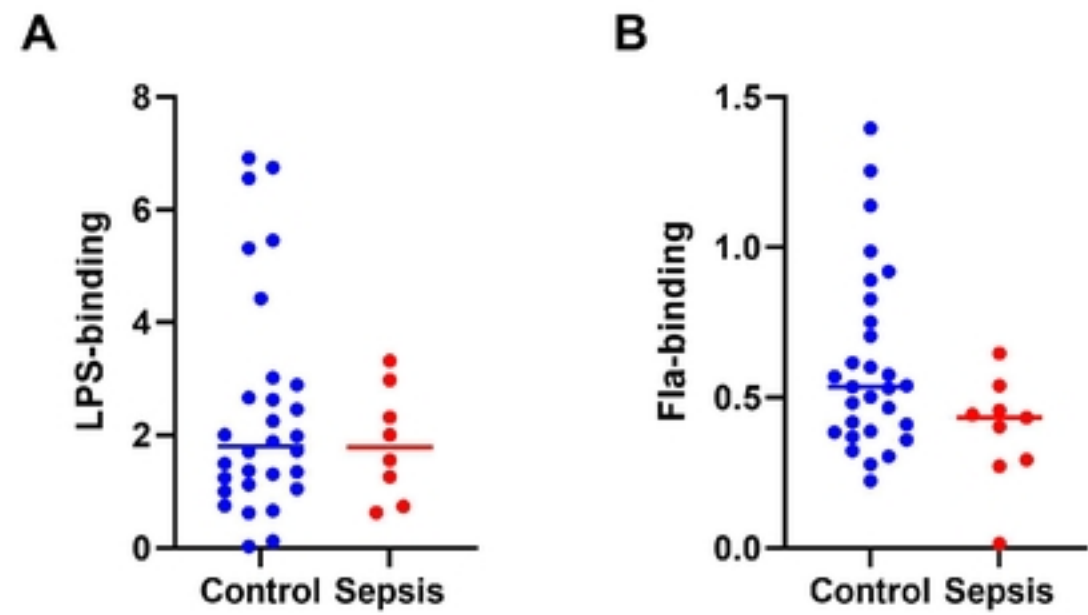

Fig S4

Figure S5

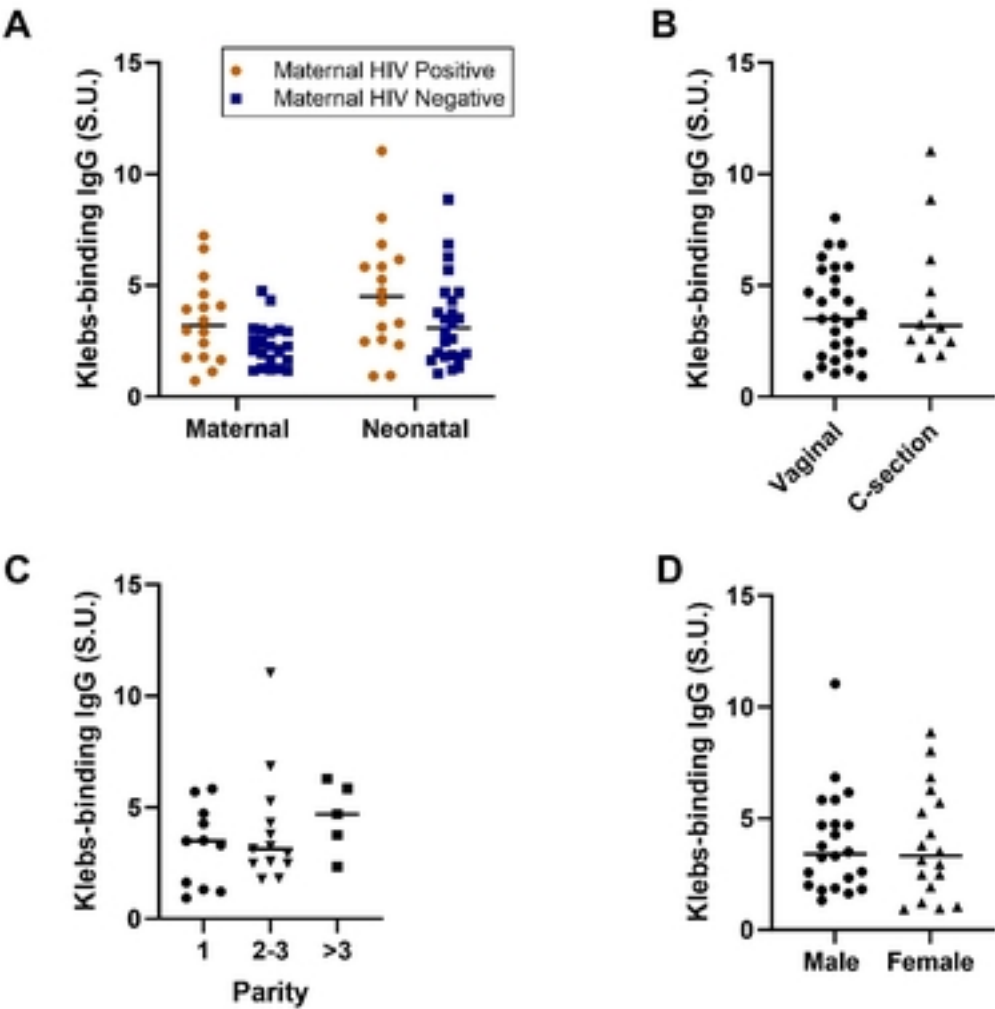

Fig S5

Supplement: Supplement 1 — Supplementary Figure 1. Immunoglobulin levels over time in cases compared to controls. A) Total IgG concentration recovered from each sample as a function of time interval between sample collection and extraction in sepsis cases compared to controls. B) Relative amount of Kleb-IgG in each DBS sample as a function of time interval between sample collection and extraction. Standard Unit (SU). Slopes were compared by analysis of covariance. Supplementary Figure 2. Levels of IgA in maternal and neonatal DBS samples. IgA level was determined using standard human ELISA from randomly selected paired maternal and neonatal samples. Mann-Whitney U test. **p<0.01. Supplementary Figure 3. Correlation between maternal Kleb-IgG and neonatal Kleb-IgG levels. A) Kleb-IgG levels for mother-neonate dyads with sepsis, simple linear regression, p=0.32. B) Kleb-IgG levels for mother-neonate dyads with sepsis, simple linear regression, p<0.01. Supplementary Figure 4. Anti-LPS IgG and anti-flagellin IgG in neonates with and without sepsis. A) ELISA comparing anti-LPS IgG in neonates with sepsis vs. controls. All units are normalized to the standard unit, defined as the amount of anti-LPS IgG in 50 ng of reference adult serum. B) ELISA comparing anti-flagellin IgG in neonates with sepsis vs. controls. Mann-Whitney U test. Supplementary Figure 5. Kleb-IgG level by maternal HIV status, neonatal sex, delivery mode, and maternal parity. A-D) Kleb-IgG binding comparison by the indicated clinical variables. Mann-Whitney U test or Kruskal-Wallis test. [file NIHPP2024.05.28.24308042v1-supplement-1.pdf]
